# Atypical Endometrial Hyperplasia and Concurrent Cancer: A Comprehensive Overview on a Challenging Clinical Condition

**DOI:** 10.3390/cancers16050914

**Published:** 2024-02-24

**Authors:** Luca Giannella, Camilla Grelloni, Marco Bernardi, Camilla Cicoli, Federica Lavezzo, Gianmarco Sartini, Leonardo Natalini, Mila Bordini, Martina Petrini, Jessica Petrucci, Tomas Terenzi, Giovanni Delli Carpini, Jacopo Di Giuseppe, Andrea Ciavattini

**Affiliations:** Woman’s Health Sciences Department, Gynecologic Section, Polytechnic University of Marche, 60123 Ancona, Italy; lucazeta1976@libero.it (L.G.); c.grelloni@pm.univpm.it (C.G.); m.bernardi@pm.univpm.it (M.B.); camilla.cicoli@pm.univpm.it (C.C.); f.lavezzo@pm.univpm.it (F.L.); g.sartini@pm.univpm.it (G.S.); l.natalini@pm.univpm.it (L.N.); m.bordini@pm.univpm.it (M.B.); martina.petrini@pm.univpm.it (M.P.); j.petrucci@pm.univpm.it (J.P.); t.terenzi@pm.univpm.it (T.T.); giovanni.dellicarpini@ospedaliriuniti.marche.it (G.D.C.); jacopo.digiuseppe@ospedaliriuniti.marche.it (J.D.G.)

**Keywords:** atypical endometrial hyperplasia, endometrial cancer, risk factors, endometrial sampling methods, lymph node status assessment, sentinel lymph node technique

## Abstract

**Simple Summary:**

Managing atypical endometrial hyperplasia (AEH) places the gynecologist in a challenging clinical situation. Therapeutic options include conservative or definitive treatment. The main concern is the possibility of concurrent endometrial cancer (EC). This occurrence can lead to undertreatment, whether in the case of conservative or surgical therapy. Therefore, the current literature has worked to identify a better diagnostic therapeutic work-up by focusing on variables predictive of occult cancer, the best preoperative endometrial sampling method, and the possibility of lymph node status assessment for women undergoing surgery. The present review aims to provide a complete overview of all these aspects to summarize the most relevant studies on these topics and better define the clinical management of women with AEH.

**Abstract:**

The present review regarding atypical endometrial hyperplasia (AEH) focused on the main debated factors regarding this challenging clinical condition: (i) predictive variables of occult endometrial cancer (EC); (ii) the rate of EC underestimation according to different endometrial sampling methods; and (iii) the appropriateness of lymph node status assessment. When cancer is detected, approximately 90% of cases include low-risk EC, although intermediate/high-risk cases have been found in 10–13% of women with cancer. Older age, diabetes, high BMI, and increased endometrial thickness are the most recurrent factors in women with EC. However, the predictive power of these independent variables measured on internal validation sets showed disappointing results. Relative to endometrial sampling methods, hysteroscopic endometrial resection (Hys-res) provided the lowest EC underestimation, ranging between 6 and 11%. Further studies, including larger sample sizes of women undergoing Hys-res, are needed to confirm these findings. These data are urgently needed, especially for female candidates for conservative treatment. Finally, the evaluation of lymph node status measured on 660 of over 20,000 women showed a lymph node positivity of 2.3%. Although there has been an increase in the use of this procedure in AEH in recent years, the present data cannot recommend this option in AEH based on a cost/risk/benefit ratio.

## 1. Introduction

Atypical endometrial hyperplasia (AEH) is a challenging clinical condition, given that concomitant endometrial cancer (EC) may be present in 40% of cases [1,2]. Although the majority of occult ECs are at low risk, the rate of intermediate/high-risk EC cases is not negligible and, therefore, needs major surgical staging [3,4,5,6,7].

On the other hand, conservative therapy is a viable option in cases of women who still wish to become pregnant [6]. Therefore, an accurate diagnosis is of pivotal importance.

The main debated topics regarding the proper diagnosis and management of AEH are represented by the predictive factors of occult EC, the endometrial sampling errors according to different methods, and the need for possible evaluation of the lymph node status in women with AEH deserving of more radical surgery.

This review aims to comprehensively report evidence on the abovementioned debated issues, summarizing all these aspects to provide a helpful tool for clinical practice regarding the correct diagnostic and therapeutic work-up of women with AEH. At the same time, it provides insights and future directions for further research.

## 2. Atypical Endometrial Hyperplasia

### 2.1. Risk Factors

Endometrial hyperplasia (EH) is a premalignant lesion characterized by the hyperproliferation of endometrial cells, particularly the stromal glandular component, caused by estrogenic action [8]. The main risk factors leading to endometrial hyperplasia (EH) are almost identical to those already known to be associated with EC. Indeed, the incidence of EH and EC is mostly a prerogative of advanced age, which correlates with the increase in body weight and the decline in fertility [9]. Other known risk factors, such as nulliparity, infertility, early menarche, and late menopause, are all related to a state of prolonged endogenous hyperestrogenism [10].

Two typical patients are particularly at risk of developing EH: a woman with polycystic ovarian syndrome (PCOS), characterized by chronic anovulation linked to a hyperandrogenic status, and the obese peri-post-menopausal patient, due to the increase in estrogenic production by fat cells [11]. In a systematic review and meta-analysis, a strong association between PCOS and EC has been observed: PCOS would increase the probability of developing EC by three times [12]. Regarding obesity, compared to women of average weight, those with a BMI between 25 and 29 have a 2.3-fold increased risk of developing EH (95% CI: 0.7, 7.9), those with a BMI between 30 and 39 have a 3.7-fold increased risk (95% CI: 1.0, 13.8), and finally, the risk increases 13-fold in those with a BMI ≥ 40 (95% CI: 1.9, 86.9) [13]. Moreover, excess adipose tissue promotes a chronic proinflammatory status, which stimulates the release of cytokines that are also involved in developing EH and EC [10]. In a patient diagnosed with EH, it is also advisable to exclude the presence of tumors secreting sex hormones, such as granulosa cell tumors of the ovary, which leads to an anomalous endogenous release of estrogens with chronic hyperstimulation of the endometrium [14].

Chronic hyperestrogenism may also be exogenous/iatrogenic: it is known that prolonged estrogenic therapy with increasing doses not opposed by an adequate intake of progestins, especially in post-menopausal women, carries a rising risk of developing EH and EC [14]. Tamoxifen is the most widely used among selective estrogen receptor modulators (SERMs) against breast cancer in both pre- and post-menopausal women. However, it can lead to endometrial thickening and polyps, all related to EH and EC over time [10]. Even though most cases of EH and EC are isolated, some genetic diseases significantly increase the risk, such as Lynch Syndrome, also known as hereditary non-polyposis colonic cancer (HNPCC), caused by a mutation in one of the genes involved in the DNA repair system (MLH1, MSH2, MSH6, PMS2, and EP-CAM) [9]. A study reviewed the current risk of developing EC in a population of women affected by Lynch syndrome, and it ranges from 30 to 45% (20–30 times higher compared to the general population) [15]. Table 1 reports the risk factors associated with EH.

### 2.2. Histological Classification of Endometrial Hyperplasia

Historically, the 1994 WHO classification for EH distinguished four subcategories: simple endometrial hyperplasia without atypia; complex endometrial hyperplasia without atypia; simple endometrial hyperplasia with atypia; and complex endometrial hyperplasia with atypia. This classification was based only on descriptive histological findings and showed low agreement and reproducibility rates. In addition, two of the four categories (simple endometrial hyperplasia with atypia and complex endometrial hyperplasia without atypia) were poorly represented in the general population, so their biological meaning was doubtful [16,17].

In 2000, the International Endometrial Collaborative Group proposed a new classification system (EIN, 2000) based on quantitative morphological criteria. The idea was to simplify the previous classification by dividing the classes of EH into two categories: benign (Endometrial Hyperplasia) and premalignant (Endometrial intraepithelial neoplasia, EIN) to increase its reproducibility [18].

In 2003, the WHO accepted the EIN system as an alternative to the WHO 1994 classification. EIN 2000 classification showed better diagnostic performances than the 1994/2003 WHO classifications and represented the basis for drafting the 2014 WHO classification [16].

The 2014 WHO classification distinguished EH into two categories: hyperplasia without atypia and atypical endometrial hyperplasia (AEH)/EIN [19]. The 2014 WHO classification has proven to have a more robust prognostic power, excellent reproducibility, and alignment with therapeutic options [20]. For these reasons, this subdivision was also maintained in the most recent WHO classification version of 2020, which, for the first time, recommended the inclusion of immunohistochemical biomarkers into the diagnostic workup for AH/EIN [21]. Table 2 shows the EH/AEH classification over time.

The terms AEH/EIN describe the simultaneous changes of epithelial cytology and an increased number of endometrial glands in comparison with the stroma within a definable endometrial region distinct from the surrounding endometrium or from entrapped normal glands [21]. The stroma appears reduced about the glandular component, proliferating crowdedly [22,23] and presenting cells with cytologic atypia. From a molecular point of view, it has emerged that AEH is associated with mutations acquired by pathological cells. Among these, the most common are mutations in PTEN, KRAS, PI3KCA, CTNNB1, and/or ARID1A and microsatellite instability (Lynch syndrome) [21]. Table 3 reports the classification of EH according to WHO 2014 and 2020.

### 2.3. Role of Immunohistochemistry in Diagnostics

An accurate diagnosis of AEH/EIN is still a challenge for pathologists as sometimes subjective histological criteria or different classifications are adopted between different institutions. In addition to the essential morphological criteria, the WHO 2020 classification assesses that a deficient expression of PTEN, PAX2, or Mismatch Repair (MMR) at the immunohistochemical analysis is a desirable criterion for the diagnosis of AEH/EIN [21]. This concept suggests an immunohistochemistry panel every time a histological diagnosis of AEH/EIN is performed, but WHO does not specify which and how many markers should be analyzed.

Recently, new immunohistochemical markers of AEH/EIN have been proposed, such as the loss of β-catenin and Arid1a and the upregulation of YTHDF2 [25].

American research systematically compared the diagnostic performances of six immunohistochemical markers of AEH/EIN (PAX2, PTEN, β-catenin, Arid1a, Mlh1, and p53) both alone and in association with each other. A panel of only three markers (Pax2, Pten, and β-catenin) acted as a valid adjuvant diagnostic tool for the histological diagnosis of AH/EIN, with at least one of the three markers being deficient in 92.8% of AEH/EIN. The same focused panel accurately detected AEH/EIN on endometrial polyp lesions [26,27].

Some authors proposed some immunohistochemical markers as indicators of progression risk to EC. In particular, the expression of β-catenin, especially in the nuclear zone, reflects the mutation of the CTNNB1 gene and could represent a good prognostic tool for EC development [28].

PTEN loss in AEH specimens is associated with a higher risk of co-existent cancer (over 50%), but it shows weak prognostic accuracy. PTEN deficiency could be helpful in borderline cases for the choice of therapy or could indicate closer surveillance during follow-up [29]. A British research group recently created a panel of three markers (HAND2, PAX2, and PTEN) that showed good malignant progression risk stratification [30].

However, more extensive prospective studies using immunohistochemical factors and blood biomarkers are required before including these tools in routine clinical practice.

### 2.4. Progression Risk

EH with atypia has a cumulative risk of progression to endometrial cancer that increases from 8.2% in the first four years to 12.4% at nine years up to 27.5% (95% CI 8.6% to 42.5%) at nineteen years from diagnosis [31]. A recent systematic review examining ten papers concluded that the overall risk of evolution to EC for endometrial hyperplasia without atypia is 2.6% per year. For AEH, it is equal to 8.2% per year. However, studies have shown much heterogeneity in data analysis, likely because of the lack of univocal histological criteria to distinguish AEH from EC among pathologists [32].

### 2.5. Management

#### 2.5.1. Surgical Management of Atypical Endometrial Hyperplasia

In the management of patients affected by AEH, it is necessary to consider women’s age, family history, the presence of any comorbidities (i.e., Lynch syndrome), and reproductive desire [9].

In patients affected by AEH, given the high risk of progression and the risk of having concomitant EC, the treatment of choice is total hysterectomy [31,33]. AEH is associated with a risk of underlying cancer (i.e., endometrial cancer, ovarian cancer). For this reason, the surgical approach should also include evaluation of the adnexa and other structures within the pelvis to exclude signs of previous and intraoperative invasion [22]. If feasible, the laparoscopic approach is recommended. A recent review has demonstrated that regarding early-stage EC, laparoscopic surgery is associated with similar results in terms of overall and disease-free survival, reduced morbidity and reduced hospitalization times, and similar quality of life compared to laparotomic surgery [34]. No scientific evidence supports the execution of the extemporaneous histological examination of surgical specimens [22].

#### 2.5.2. Conservative Management of Atypical Endometrial Hyperplasia

In patients who are not eligible for surgery due to low-performance status or in women who express the desire to become pregnant, it is possible to propose conservative treatment (fertility-sparing treatment) with the objectives of preventing progression to carcinoma, preventing invasive disease, and restoring the endometrium to its physiological functions [35].

In the case of fertility-sparing treatment, the patient must be informed that they may have co-existing ovarian cancer with EH (4%) and that the risk of progression of EC to higher stages than stage I is approximately 2%. The risk of metastatic disease and death is approximately 0.5% [36].

Progestin therapy represents the basis of conservative treatment of EH regardless of histological classification. The results of a systematic review of 24 studies with 1001 patients with AEH showed that progesterone-based IUD induces more significant regression than oral progestins (90% vs. 69%) [37].

In a recent systematic review involving 2268 patients, LNG IUS was associated with higher regression rates than medroxyprogesterone acetate (MPA) (RR 1.30, 95% CI 1.16–1.46) in patients with EH. The LNG IUS may represent the first-line progestin agent for women with EH, and the association with MPA may further improve its effectiveness [38].

Identifying any disease progression early is of primary importance during conservative treatment, which requires close follow-up. The most recent guidelines relating to treating EH recommend that follow-up should be carried out through endometrial biopsies [39,40].

Given the high risk of recurrence, risk of progression, and concomitant endometrial cancer, authors recommend performing endometrial biopsies every 3 to 6 months [9,40] to evaluate the response to treatment. After the first follow-up, it is recommended to execute a second endometrial biopsy 3–6 months later [39] as histological disease regression is defined after two consecutive negative endometrial biopsies [40]. If there is no response after 9–12 months of hormonal therapy, it is advisable to discuss with the patient the possibility of definitive surgical treatment [39,40].

In recent years, a growing interest in predictive factors for the success of conservative treatment in AEH/EIN has spread. A systematic review assessed the predictive performance of over forty immunohistochemical markers, including estrogen receptors (ER), progesterone receptors (PR), PTEN, PAX2, MMR, COX2, Ki-67, and others. Although several studies demonstrated that high expression of ER and PR could represent a good marker of response to hormonal therapy (in particular to LNG IUS), their predictive value still needs to be improved for actual clinical usefulness. Among the progesterone isoforms, PRB appeared to be the most promising in the pretreatment evaluation [41].

Post-treatment immunohistochemical markers can be assessed in endometrial specimens during the follow-up to change therapy if needed. Good responders showed a down-regulation of ER and PR, while expression of Nrf2 and AKR1C1 was associated with poor response due to progesterone resistance [41]. Other biomarkers have been proposed, but further results are needed to create valid immunohistochemical panels for response prediction and therapy management.

## 3. Atypical Endometrial Hyperplasia and Concurrent Endometrial Cancer: The Role of Predictive Factors and Endometrial Sampling Methods

The risk of developing EC in patients with AEH is approximately 40% [42]. However, the literature estimates that patients diagnosed with EH undergoing hysterectomy have concomitant EC in about 20–48% of the cases [1,2,42]. These data demonstrate the difficulty of correct preoperative diagnosis and difficult anatomopathological interpretation of the specimen.

In most cases of concurrent EC, the prognosis is good. According to a retrospective study conducted from 2010 to 2020, 99% of patients diagnosed with concurrent EC had FIGO stage IA, an endometrioid histotype, and, in most cases, a lesion <2 cm in diameter [43]. In a recent systematic review in which 503 patients diagnosed with EH underwent hysterectomy, the incidence of concurrent EC was 45%, of which 199 patients had FIGO stage 1A (89.2%) [44]. However, in a retrospective cohort study that included 169 women diagnosed with EIN, 82 were diagnosed with concurrent EC. Of these, about 10% had a high-grade tumor, and lymph node involvement was approximately 3–7% [42]. For this reason, recent studies have focused on identifying predictive risk factors for concurrent EC diagnosis. In the literature, many studies agree on the impact that age, obesity, and endometrial thickness have on the probability of having concurrent EC [42,43,45,46].

Preoperative endometrial thickness measured by transvaginal ultrasound is a predictive factor for EC. According to the study by Vetter et al., patients with endometrial thickness ≥ 2 cm have an approximately 4-fold higher risk of being diagnosed with concurrent EC. Moreover, 44% of these patients present clinicopathological features suspicious of lymph node involvement (Mayo Criteria) [42]. These data are confirmed by a recent retrospective study aimed at framing patients who could benefit from sentinel node study. In that study, an endometrial thickness ≥ 15 mm is associated with having Mayo Criteria worthy of lymph node status assessment (crude RR 2.5, 95% CI 1.2 to 5.2) [43].

In a retrospective study analyzing 168 patients diagnosed with EH undergoing hysterectomy, four independent risk factors for the diagnosis of EC were identified. These include age ≥ 60 years (age 40–59, OR 3.07, 95% CI 1.18–7.97, *p* = 0.021; and age ≥ 60, OR 6.65, 95% CI 1.75–25.3, *p* = 0.005); BMI > 35 kg/m^2^ (OR 2.32, 95% CI 1.09–4.93, *p* = 0.029); presence of diabetes mellitus (OR 2.51, 95% CI 1.16–5.39, *p* = 0.019); and diagnosis of AEH (OR 9.01, 95% CI 1.09–74.6, *p* = 0.042). Moreover, the risk of concurrent EC increases as the number of risk factors rises (none 0%, one risk factor 7.0%, two risk factors 17.6%, three risk factors 35.8%, and four risk factors 45.5%, *p* < 0.001) [45]. In contrast, parity, use of Tamoxifen, hormone therapy, or the presence of abnormal uterine bleeding are not statistically significant risk factors [47]. According to a meta-analysis aimed at analyzing cancer incidence in correlation with BMI, an increase of 5 kg/m^2^ is associated with an approximately 1.6-fold increased risk of having EC. This is caused by the production of estrogens by the adipose tissue and the subsequent action of these hormones on the growth of the endometrium [48]. Although all these independent variables were associated with EC in women with a preoperative diagnosis of AEH, a recent study including regression analysis and artificial intelligence showed several predictive models achieving unsatisfactory results in the validation set regarding the predictive power of patient characteristics [49].

Another aspect debated in the literature concerns the most accurate endometrial sampling method for diagnosing endometrial pathologies. Dilatation and curettage (D&C) have been the most used method in the past years. In most curettage procedures, less than half of the uterine cavity is sampled; in this way, part of the lesion could remain inside the cavity or not be analyzed [50,51]. This limitation led to comparison with other endometrial sampling methods, for example, hysteroscopic guided biopsy (HSC-bio) or hysteroscopic endometrial resection (HSC-res). Hysteroscopic methods can directly visualize the altered endometrium and perform a targeted biopsy. In a meta-analysis that included 39 studies, the pipelle endometrial sampling method was found to have a sensitivity for diagnosing EC of 91% in pre-menopausal patients and 99.6% in post-menopausal patients. However, it is a blind method with the abovementioned limitations [52]. Furthermore, most of these studies had dilation and curettage (D&C) as histology reference standards [47]. Several publications have reported hysteroscopic resection as the most accurate diagnostic method for detecting endometrial pathologies. More tissue is removed during hysteroscopic resection compared to the other two methods (D&C, HSC-bio). In a retrospective study conducted on 208 women with AEH and undergoing hysterectomy between January 2000 and December 2017, it was demonstrated that hysteroscopic resection had the lowest percentage of EC underestimation: HSC-res = 11.6%; HSC-bio = 19.5%; D&C = 35.3% [47]. These results are in agreement with a systematic review that considered 27 studies, with a total of 1106 patients, in which HSC-res was found to be the most accurate endometrial sampling method with an underestimation percentage of 5.8% (95% CI, 0.8–31.7) [50]. However, it should be underlined that hysteroscopic resection is an invasive technique that requires being performed in the operating room with anesthesia and hospitalization for the patient.

Therefore, the safest sampling method is still debated in the literature; the choice should consider predictive factors for EC, and an endoscopic procedure should likely be preferred, especially when conservative treatment is chosen. Table 4 reports studies showing the EC rate in women with AEH and its correlation with endometrial sampling methods. Table 5 shows the predictive factors of concurrent EC in AEH.

## 4. Data on Lymph Node Status Assessment in Atypical Endometrial Hyperplasia (AEH)

To summarize the current state of the art, we performed research using the electronic medical databases Pubmed and Scopus, combining the following words: (“endometrial atypical hyperplasia” or “endometrial intraepithelial neoplasia”) and (“sentinel lymphadenectomy” or “sentinel lymph node” or “lymph node dissection”).

Twelve articles in the English language concerning patients with AEH or Endometrial Intraepithelial Neoplasia (EIN) who underwent surgical treatment combined with lymph node staging were included.

As shown in Table 6, the selected articles were all retrospective studies and considered a total of 22,201 women [3,4,43,58,59,60,61,62,63,64,65,66]. The rate of occult endometrial carcinoma in patients with a preoperative diagnosis of AHE ranged from 27.2 to 53.3%, consistent with the previous literature [1,2].

Some studies systematically subjected all patients to lymph node evaluation, while others selected high-risk patients based on clinical characteristics, frozen section analysis, or clinic criteria (Mayo, Gog 99, and ESGO classification).

Despite the lack of precise guidelines on lymph node staging in patients with AEH, in research by Dioun et al., from 2012 to 2018, the rate of SLNB increased from 0.8% to 14%, while that of pelvic lymphadenectomy rose from 5.7 to 6.4% (*p* < 0.001) [60].

The radiotracer substances used for SLNB were indocyanine green, technetium-99, and isosulfan blue. Histological analysis of lymph nodes was available for 660 cases. The positivity rate among patients subjected to lymph node studies varied from 0% to 10%, with a mean of 2.36%.

El-Achi et al. conducted a retrospective study with 29 patients with a preoperative diagnosis of AEH and a rate of EC at the final histology of 41.9%. Thirteen patients had sentinel lymph node examinations, all of which were negative [61].

The study of Touhami et al. evaluated the risk of occult EC and lymph node positivity in patients with a preoperative diagnosis of AEH and atypical endometrial hyperplasia “cannot rule out carcinoma” (AEHc) [4]. This expression is not part of the official nomenclature and was coined by Longacre in 1995 to describe histologic cases with no clear evidence of EC but only suspicious histologic features, such as extensive glandular complexity, suggesting a disease more severe than AEH [67]. A total of 70 patients diagnosed with AEH and 50 diagnosed with AEHc were included. Sixty-four patients had a final diagnosis of EC, of which 66% had an initial diagnosis of AEHc, while 44% had an initial diagnosis of AEH. A study including the SLNB technique was systematically performed. Approximately 8% of patients with an initial diagnosis of AEHc and a final diagnosis of EC had lymph node positivity (4/50). In contrast, no pathological lymph node was found in the patients with a preoperative AEH diagnosis and a final EC diagnosis. Therefore, the authors concluded that a preoperative diagnosis of AEHc was a valid parameter to identify patients who may benefit from lymph node sampling. However, more prospective data and official histological terminology are needed [4].

Abt et al. tried identifying possible clinical indicators of positive lymph nodes during hysterectomy in AEH. In this study, including 378 women with AEH, the EC rate was 27.2% with 31% high-risk carcinoma at the final histological specimen, according to the Mayo Clinic Criteria. Lymph node sampling was performed in 10 patients, 50% of whom had the SLNB technique, while the rest had bilateral pelvic lymphadenectomy. No pathological lymph nodes were found. After re-evaluating patients’ characteristics, two variables correlated with the risk of concomitant EC: older age and increased endometrial thickness [43].

While Vetter had previously demonstrated that endometrial thickness >20 mm entailed a 4-fold greater risk of concomitant EC, Abt et al. showed firstly that endometrial thickness >15 mm increased the risk of concomitant EC and secondly that these women were more likely to meet high-risk Mayo Criteria, with an RR of 2.2 [42,43]. Therefore, the author concluded that an endometrial thickness ≥15 mm can be considered a criterion to predict the need for lymph node evaluation using SLNB. However, studies are needed to confirm this [43].

Mueller et al. analyzed data from 221 cases of EIN/AEH undergoing surgical treatment. Of these, 99 patients (about 44.7%) received a diagnostic upgrade to EC, and 10% received adjuvant treatment for stages of disease higher than IA1, according to the FIGO classification. One hundred and sixty-one patients received lymph node evaluation through the SLNB technique. Lymph node positivity was found in less than 1% of cases (about 0.5%). Despite the low incidence of lymph node pathology in such a large sample of patients, the authors suggested that SLNB should be proposed for several reasons, including the high incidence of occult EC following the preoperative diagnosis of EIN/AEH and the need for a complete staging in high-grade carcinoma. Data showed that SLNB does not increase operative time nor intra- and extra-operative complications and reduces the concerns of patients with a diagnostic upstaging. Despite these advantages, the SLNB increases procedural costs [66].

Furthermore, Dioun in 2021 demonstrated that SLNB, in addition to laparoscopic hysterectomy, increases the procedural cost by about 28%, compared to hysterectomy alone, while it decreases the procedural cost by about 6% compared to bilateral pelvic dissection. Interestingly, no difference in terms of cost in women undergoing robotic surgery versus laparotomic/laparoscopic surgery was found [60].

The retrospective study by Sullivan et al. included 151 patients with pre-invasive endometrial lesions (AEH/EIN). The aim was to determine how many of these women met Mayo Criteria for lymphadenectomy to assess the utility of universal SLNB. Of the seven patients who met Mayo Criteria, one had grade 3 disease, seven had a tumor size > 2 cm, one had a myometrial invasion by disease >50%, and three had lymph nodes assessment with zero cases of metastases. A total of 7 patients underwent lymph node assessment (three SLNB and four pelvic lymphadenectomy), and only six of them had a final diagnosis of EC. The only factor found to be predictive of meeting Mayo Criteria was age ≥ 55 years (85.7% vs. 31.8%, *p* = 0.007). In this population, 51 cases of endometrial cancer (36.2%) were diagnosed, 7 (13.7%) of which met Mayo Criteria for high-risk EC. Tamoxifen therapy, type 2 Diabetes Mellitus, and Lynch Syndrome were found to be predictors of EC. The authors admitted to performing systematic intraoperative frozen sections to identify EC in their institution. However, this technique showed low accuracy, with a sensitivity of 25% to predict a final diagnosis of EC. The authors conclude by stating that their data suggest that SLNB is not warranted in women diagnosed with AEH/EIN [62].

Whyte et al. in 2010 subjected 88 patients with AEH to surgery: 63 (71.6%) of them underwent pelvic lymphadenectomy, while 4 (4.5%) patients underwent pelvic + paraaortic lymphadenectomy. Just 1 of the 67 patients who underwent lymph node evaluation had positive lymph nodes (1.4%), but surgical staging influenced treatment in 7/25 (28%) of patients finally diagnosed with EC [3].

Similar conclusions can be drawn from the study by Matanes et al. (2023), which found 2/157 (1.2%) cases of positive lymph nodes in patients with EAH. Lymph node evaluation provided crucial information in 14 patients (8.3%) referred for adjuvant treatment [64].

Data extracted from Taşkın et al. (2017) are consistent with the ones mentioned above. They found 2/37 (5.4%) cases of lymph nodal metastases after performing 28 pelvic lymphadenectomy and 9 pelvic + paraaortic lymph nodal staging in patients diagnosed with AEH. They also noticed that in many cases (17/40, 42.5%), lymph nodal evaluation could provide pivotal information over adjuvant treatment [59].

Capozzi et al. registered a lymph node positivity of 0.7% (1/54). At the same time, 9.9% (15/152) of patients with AEH received a final diagnosis of endometrial carcinoma at intermediate or higher risk, confirming the elevated number of patients who could obtain information from nodal assessment [63].

In 150 patients with AEH, Costales noticed that 1 of 10 (10%) patients who underwent pelvic lymphadenectomy had positive lymph nodes [58]. They tried to evaluate the risk of lymph node involvement on the whole cohort of patients using GOG-33 criteria, Bendifallah nomogram, and AlHilli nomogram, estimating the risk between 1.6 and 2.1% [68,69,70,71].

No studies specified whether lymph node ultra-staging was performed, even if published in different years. This fact could lead to an underestimated rate of lymph node involvement.

The studies considered in our revision showed, in patients with AEH, a risk of lymph node involvement in about 2–3% of cases. What emerges, however, is the incidence of intermediate/high-risk endometrial carcinoma in patients with a previous diagnosis of AEH, who may require lymph node evaluation to better define adjuvant treatment strategies. Nowadays, no risk stratification offers an accurate prediction concerning the risk of nodal involvement before surgery, and a frozen section during a hysterectomy seems unreliable due to its low sensitivity.

Moreover, we must consider the weaknesses of the included studies because of their retrospective nature, the need for predictive factor assessment of lymph node positivity, heterogeneous populations, and non-standardized criteria for performing lymph node assessment.

## 5. Conclusions

As emerged from this comprehensive review, the rate of occult cancer can be up to 40%, and of these cases, approximately 10–13% include high-risk EC. Several independent variables, such as older age, obesity, and diabetes mellitus, have shown association with this occurrence. However, their predictive power on internal validation sets has not reported satisfactory results. Although specific patient characteristics are recurrent in women with EC, their discriminating power is not optimal in validation analyses [40]. Other factors (e.g., genotypic variables) likely need to be included in risk stratification to move towards more personalized medicine.

Based on data in the literature, the studies regarding the preoperative endometrial sampling methods in women with AEH compared blind and targeted biopsies. Currently, the endoscopic HYS-res method offers the lowest rate of EC underestimation. This information is crucial, especially for women candidates for conservative treatment. However, further studies, including larger sample sizes, must confirm these data.

Based on the data above, we reported how lymph node status assessment has increased over the years in women with AEH, including the sentinel lymph node technique. However, the reported studies provided an average prevalence of lymph node positivity of 2.3%. So far, these numbers cannot suggest using such a procedure based on a cost/risk/benefit assessment.

## Figures and Tables

**Table 1:** Risk factors associated with EH [10,11].

**Individual Factors**	Older age Family history Genetic factors (e.g., SNPs, PTEN, K-ras, β-catenin, PIK3CA mutations, deletions on the short arm of chr-8, MSI).
**Reproductive phase**	Menopause Early menarche/late menopause Perimenopause Nulliparity Infertility
**Comorbidities**	Obesity Diabetes mellitus Polycystic ovarian syndrome (PCOS) Functional tumors (e.g., granulosa cells) Lynch syndrome
**Iatrogenic**	Tamoxifen therapy Hormone replacement therapy with only Estrogen Exogenous estrogen exposure

**Table 2:** Historical changes of EH/AEH according to WHO and EIN classifications.

WHO 1994/WHO 2003	EIN 2000	WHO 2014/WHO 2020
Simple endometrial hyperplasia without atypia	Endometrial hyperplasia	Hyperplasia without atypia
Complex endometrial hyperplasia without atypia		
Simple endometrial hyperplasia with atypia	Endometrial intraepithelial neoplasia	Atypical hyperplasia/EIN
Complex endometrial hyperplasia with atypia		

**Table 3:** Classification of EH.

Classification (According to WHO 2014 and 2020 [16,21])	Genetic Profile	Essential Histological Features
Hyperplasia without atypia	No characteristic mutational component.	Increased endometrial gland to stroma ratio Tubular branching and/or cystically dilated glands resembling proliferative endometrium Uniform distribution of nuclear features
Atypical hyperplasia/endometrioid intraepithelial neoplasia	Microsatellite instability (including Lynch syndrome), PAX2 inactivation, PTEN, KRAS, PI3KPCA, CTNNB1, ARID1A [16,24]	Crowded glandular architecture and altered epithelial cytology distinct from the surrounding endometrium and/or entrapped non neoplastic glands [16] Must exclude common mimics (endometrial polyps, dyssynchronous phase endometrium) [16]

**Table 4:** The most relevant studies showing concurrent EC in women with EH and correlation with endometrial sampling methods.

Author, Year	Sample Size (n)	Rate of Endometrial Cancer (%)	Rate of Low-Risk Endometrial Cancer (%)	Rate of Intermediate/High-Risk Endometrial Cancer (%)	Rate of EC in D&C	Rate of EC in HSC-bio	Rate of EC in HSC-res
Bourdel et al. 2016 [50]	1106	(31%)			32.7%	45.3%	5.8%
Erdem et al. 2018 [46]	227	(25.1%)	50/57 (87.7%)	7/57 (12.3%)			
Trimble et al. 2006 [33]	115	(39.1%)					
Rakha et al. 2012 [53]	2571	(37%)					
Giannella et al. 2020 [47]	208	(23.6%)	37/49 (75.5%)	10/49 (20.4%) 2/49 (4%)	35.3%	19.5%	11.6%
Giede et al. 2008 [54]	70	(35.7%)	21/25 (84%)	4/25 (16%)			
Miller et al. 2008 [55]	48	(37.5%)					
Whyte et al. 2010 [3]	88	(28.4%)	20/25 (80%)	5/25 (20%)			
Hahn et al. 2010 [56]	126	(10.3%)	13 (100%)				
Leitao et al. 2010 [57]	197	(34%)	56/67 (83.6%)	11/67 (16.4%)			
Vetter et al. 2020 [42]	169	(48.5%)	(90%)	(10%)			
Vieira-Serna et al. 2023 [44]	503	(44.5%)	199/224 (89%)	25/224 (11%)			
Travaglino et al. 2019 [8]	960	(45.5%)					
Matsuo et al. 2015 [45]	211	(20.4%)	36/43 (83.7%)	7/43 (16.3%)			
Abt et al. 2022 [43]	378	(27%)	98/103 (95%)	5/103 (5%)			

**Table 5:** Predictors of concurrent EC in women with EH according to the most relevant studies.

Author, Year	BMI	Age	Endometrial Stripe Thickness	Hypertension	Diabetes Mellitus	Parity
Giannella et al. 2020 [47]	BMI ≥ 40 (OR 19.751, 95% CI 2.193–177.829, *p* value 0.007)	Age > 60 (OR 1.055, 95% CI 1.002–1.111, *p* value 0.039)				
Erdem et al. 2018 [46]	BMI > 30 (OR 1.0, 95% CI 0.5–2.2, *p* value 0.8082)	Age > 50 (OR 3.9, 95% CI 1.8–8.3, *p* value 0.0003)		(OR 2.6, 95% CI 1.3–5.1, *p* value 0.0022)	(OR 2.0, 95% CI 1.4–4.1, *p* value 0.0041)	Nulliparity (OR 3.5, 95% CI 1.6–8.1, *p* value 0.0029)
Vetter et al. 2020 [42]		Age ≥ 65 (OR 2.3, 95% CI 0.9–5.9, *p* value 0.08)	≥2 cm (OR 4.0, 95% CI 1.6–10.1)			
Matsuo et al. 2015 [45]	BMI ≥ 35 (OR 2.32, 95% CI 1.09–4.93, *p* value 0.029)	Age ≥ 60 (OR 6.65, 95% CI 1.75–25.3, *p* value 0.005)			(OR 2.51, 95% CI 1.16–5.39, *p* value 0.019)	
Abt et al. 2022 [43]		Older age (*p* value 0.003)	≥2 cm (OR 2.0, 95% CI 1.3–2.9)	(*p* value 0.02)		

**Table 6:** The most relevant study including women with AEH and undergoing LN status assessment.

Author, Year	Study Design	Sample Size (n)	Rate of EC (%)	Rate of Low-Risk EC (%)	Rate of Intermediate/ High-Risk EC (%)	Predictors of EC	Patients with Lymph Nodes Sampling (n, %)	Type of Lymph Node Study (n, %)	Patients with Positive Lymph Nodes; n (%)	Predictors of Positive Lymph Nodes
Whyte J.S., 2010 [3]	Retrospective	88 AEH	28.4% (25/88)	NE	NE	NE	67/88 (76.1%)	63/67 (94.0%) SPeL 4/67 (5.9%) SPeL + paraaortic	1/67 (1.4%)	NE
Costales A.B., 2014 [58]	Retrospective	150 AEH	36.7% (55/150)	NE	NE	NE	10/150 (6.7%)	10/10 (100.0%) SPeL	1/10 (10.0%)	NE
Taşkın S., 2017 [59]	Retrospective	80 AEH	50.0% (40)	NE	NE	NE	37/80 (46.2%)	28/37 (75.6%) SPeL 9/37 (24.3%) SPeL + paraaortic	2/37 (5.4%)	NE
Touhami O., 2018 [4]	Retrospective	120, of which: −70 AEH (58.3%) −50 AEHc (41.6%)	53.3% (64/120)	NE	NE	NE	120/120 (100%)	119/120 (99.2%) SLNB + SPeL 1/120 (0.8%) SPeL	4/120 (3.3%)	Preoperative diagnosis of AEHc
Dioun S., 2021 [60]	Retrospective	1026 AEH	NE	NE	NE	NE	1158/10,266 (11.2%)	620/1158 (53.5%) SLNB 538/1158 (46.4%) SPeL	NE	NE
El-Achi V., 2021 [61]	Retrospective	29 AEH	41.9% (12/29)	NE	NE	NE	13/29 (44.8%)	13/13 (100.0%) SLNB	0/13 (0%)	NE
Sullivan M.W., 2021 [62]	Retrospective	141, of which: - AEH 82 (58.2%) - EIN 59 (41.8%)	36.2% (51/141)	86.3% [Mayo Criteria]	13.7% [Mayo Criteria]	- Tamoxifen/HRT exposure, - Lynch Syndrome - Type 2 DM	7/141 (5.0%)	3/7 (42.8%) SLNB 4/7 (57.1%) SPeL	0/7 (0%)	NF
Abt D., 2022 [43]	Retrospective	378 AEH	27.2% (103/378)	69.0% [Mayo Criteria]	31.0% [Mayo Criteria]	- Older age - Increased endometrial stripes (>15 mm)	10/378 (2.6%)	5/10 (50.0%) SLNB 5/10 (50.0%) SPeL	0/10 (0%)	Endometrial stripe > 15 mm
Capozzi V.A., 2022 [63]	Retrospective	152 AEH	35.6% (54/152)	72.2% (39/54) [ESGO Risk Classification]	27.8% (15/54) [ESGO Risk Classification]	- Hypertension - AUB - Increased endometrial stripes (≥20 mm)	54/152 (33.5%)	NE	1/54 (1.8%)	NF
Matanes E., 2023 [64]	Retrospective	162 EIN	37.7% (61/162)	75.4% [GOG99 classification]	24.6% [GOG99 classification]	NF	157/162 (96.9%)	121/157 (77.0%) SLNB 36/157 (22.9%) SLNB + SPeL	2/157 (1.2%)	NF
Matsuo K., 2023 [65]	Retrospective	19,654 AEH	NE	NE	NE	NE	2158/19,654 (10.9%)	NE	NE	NE
Mueller J.J., 2023 [66]	Retrospective	221, including both AH and EIN	44.7% (99/221)	NE	NE	NE	185/221 (83.7%)	161/185 (87.0%) SLNB 24/185 (12.9%) Failed SLNB	1/185 (0.5%)	NE

EC: Endometrial Cancer; EIN: endometrial intraepithelial neoplasia; NE: not evaluated; NF: not found; HRT: Hormonal Replacement Therapy; DM: Diabetes Mellitus; AEH: Atypical Endometrial Hyperplasia; AEHc: cannot rule out cancer; SPeL: Systematic Pelvic Lymphadenectomy; SLNB: Sentinel Lymph Node Biopsy.
